# Physicochemical Characterization, Microbiological Quality and Safety, and Pharmacological Potential of *Hancornia speciosa* Gomes

**DOI:** 10.1155/2018/2976985

**Published:** 2018-06-28

**Authors:** Uilson Pereira dos Santos, Georgina S. Tolentino, Jorge Sá Morais, Kely de Picoli Souza, Leticia M. Estevinho, Edson Lucas dos Santos

**Affiliations:** ^1^Research Group on Biotechnology and Bioprospecting Applied to Metabolism (GEBBAM), Federal University of Grande Dourados, Rodovia Dourados Itahum, Km 12, 79804-970 Dourados, MS, Brazil; ^2^CIMO-Mountain Research Centre, Agricultural College of Bragança, Polytechnic Institute of Bragança, Campus Santa Apolónia, Bragança, Portugal

## Abstract

*Hancornia speciosa* Gomes is a fruit tree, commonly known as the mangaba tree, which is widespread throughout Brazil. The leaves of this plant are used in traditional medicine for medicinal purposes. Thus, the objective of this study was to perform a physicochemical characterization, identify the lipophilic antioxidants and fatty acids, and determine the microbiological quality and safety of *H. speciosa* leaves. In addition, the antioxidant, antimutagenic, and inhibitory activities of the ethanolic extract of *H. speciosa* leaves (EEHS) against enzymes related to neurodegenerative diseases, inflammation, obesity, and diabetes were investigated. Furthermore, this study aimed at assessing the *in vivo* effects of the EEHS on the glycemia of normoglycemic and diabetic Wistar rats. Physicochemical characterization was performed by colorimetry and gas-liquid chromatography with flame ionization detection (GC-FID). The total number of colonies of aerobic mesophiles, molds, and yeasts was determined. The total coliforms and *Escherichia coli* were counted using the SimPlates kit, and sulphite-reducing *Clostridium* spores were quantified using the sulphite-polymyxin-sulfadiazine agar method. *Salmonella* spp. were detected using the 1-2 Test. The antioxidant activity of the EEHS was measured by its inhibition of 2,2′-azobis(2-amidinopropane) dihydrochloride- (AAPH-) induced oxidative hemolysis of human erythrocytes. The antimutagenic activity was determined using the Ames test. The acetylcholinesterase, butyrylcholinesterase, tyrosinase, hyaluronidase, lipase, *α*-amylase, and *α*-glycosidase enzyme-inhibiting activities were assessed and compared with commercial controls. The *in vivo* effects of the EEHS were assessed using the oral glucose tolerance test in normoglycemic Wistar rats and measuring the blood glucose levels in diabetic rats. The results demonstrated physical-chemical parameters of microbiological quality and safety in the leaves of *H. speciosa*, as well as antioxidant and antimutagenic activities and inhibition of enzymes related to neurodegenerative diseases, inflammation, obesity, and diabetes. In *in vivo* assays, it was shown that the normoglycemic rats challenged with glucose overload show significantly decreased blood glucose levels when treated with the EEHS. Taken together, the results ensure the microbiological quality and safety as well as showing the contents of carotenoids and polyunsaturated fatty acids of *H. speciosa* leaves. Additionally, the antioxidant, antimutagenic, anti-inflammatory, anti-Alzheimer's disease, anti-Parkinson's disease, antiobesity, and antihyperglycemic activities of the EEHS were demonstrated.

## 1. Introduction

In different cultures worldwide, medicinal plants are used for therapeutic purposes. Although the microbiological quality of these materials is still rarely discussed, it is essential to achieve the expected pharmacological outcomes. Plant materials for medicinal purposes need to have acceptable microbial contamination levels and no deterioration or pathogenic microorganisms [1]. These basic criteria must be assessed and followed to obtain plant samples with quality, safety, and therapeutic efficacy [2, 3].

In Brazil, several plants are used in traditional medicine, including *Hancornia speciosa* Gomes (*H. speciosa*). This fruit tree, commonly known as the mangaba tree, belongs to the Apocynaceae family, and its leaves are sold as tea [4]. This species is native to Brazil and is found in the Amazonia, Caatinga, and Atlantic forests as well as the Cerrado (Brazilian savannah) biomes.

The antioxidant, antimicrobial, cytotoxic [4], anti-inflammatory [5, 6], wound-healing [6], vasodilation [7, 8], antihypertensive [9, 10], antidiabetic [11], and acetylcholinesterase-inhibiting [12] activities of *H. speciosa* leaves have been described. Previously reported phytochemical studies with *H. speciosa* leaf extracts showed a complex chemical composition. The identified chemical constituents that were related to the biological activities include L-(+)-bornesitol, quinic acid, chlorogenic acid, and kaempferol, quercetin, isoquercetin, rutin, and catechin flavonoids [4, 5, 11, 13, 14]. Recently, Bastos et al. [15] identified other phenolic compounds, including caffeic acid, protocatechuic acid isomers, epicatechin, quercetin isomers, type-B and type-C procyanidins, coumaroylquinic acid isomers, phlorizin, phloretin, eriodictyol, luteolin, and apigenin. Natural extracts and phenolic derivatives with pharmacological potential may serve as a safe and cost-effective treatment strategy as an alternative to synthetic drugs [16–19].

Plants are sources of biologically active compounds that are used by approximately 80% of the world's population, either as tea or as pharmaceutical drugs [20]. Furthermore, natural products or derivatives play a key role in the process of the development and discovery of new compounds or drugs [21]. Over the past three decades, drugs developed from natural products have been prominent because among the drugs available for treatment, 50% for Alzheimer's disease, 8% for Parkinson's disease, 27% for inflammation, 16% for obesity, and 57% for diabetes are derived directly or indirectly from natural products [21].

Thus, the objective of this study was to perform a physicochemical characterization, identify the lipophilic antioxidants and fatty acids, and determine the microbiological quality and safety of *H. speciosa* leaves. In addition, the antioxidant, antimutagenic, and inhibitory activities against enzymes related to neurodegenerative diseases, inflammation, obesity, and diabetes of the ethanolic extract of *H. speciosa* leaves (EEHS) were investigated. Furthermore, this study aimed to assess the *in vivo* effects of the EEHS on the glycemia of normoglycemic and diabetic Wistar rats.

## 2. Materials and Methods

### 2.1. Plant Material and Ethanol Extract Preparation

*H. speciosa* leaves were collected after identifying the plant and receiving permission from the Brazilian Biodiversity Authorization and Information System (Sistema de Autorização e Informação em Biodiversidade, SISBIO; number 54470-1). The plant material was collected in Dourados, Mato Grosso do Sul State (21° 59′ 41′′ south and 55° 19′ 24′′ west), Brazil, dried in a convection oven at a temperature of 45 ± 5°C, and ground in a Wiley knife mill. A voucher specimen was deposited in the herbarium of the Federal University of Grande Dourados, Mato Grosso do Sul State, Brazil, under record number 4774. The extract was prepared by macerating the plant material in 96% ethanol (1 : 10, *w*/*v*). The mixture was kept at room temperature for 14 days. Subsequently, the extract was filtered, concentrated in a rotary evaporator under vacuum (Gehaka, São Paulo, SP, Brazil), and freeze-dried. The EEHS had a final yield of 28% and was stored at −20°C in the dark. The flavonoids rutin, catechin, and isoquercetin, previously identified in the EEHS by high-performance liquid chromatography with diode-array detection-tandem mass spectrometry (HPLC-DAD-MS/MS) [4] and used in this study, were purchased from Sigma-Aldrich.

### 2.2. Physicochemical Analysis

#### 2.2.1. Ash Content

The method used to determine the ash content of *H. speciosa* leaves consisted of desiccating 250 mg of leaf samples. The test was performed in triplicate, and the result is expressed as g/100 g of sample [22].

#### 2.2.2. Total Protein Content

The protein content was determined using the Kjeldahl method (230-Kjeltec Analyzer, Foss Tecator, Höganäs, Sweden), as described by Nogueira et al. [23]. The protein concentration was determined by multiplying the value of the total nitrogen by the factor 5.75 (*P*_(protein)_ = *N*_(nitrogen)_ × 5.75). The test was performed in triplicate, and the result is expressed as g/100 g of sample.

#### 2.2.3. Total Lipids

The crude fat was determined by gravimetry after extraction with petroleum ether using an automatic Soxtec device (FOSS, Soxtec™ 2050, Höganäs, Sweden) [22]. The test was performed in triplicate, and the result is expressed as g/100 g of sample.

#### 2.2.4. Carbohydrates and Energy

The carbohydrate content of the sample was calculated by the following difference: 100 − (g proteins + g lipids + g ash). The total energy was calculated using the following equation: energy (kcal) = 4 × (g proteins + g carbohydrates) + 9 × (g lipids). The tests were performed in triplicate, and the results are expressed as g/100 g of sample for carbohydrates and as kcal/100 g of sample for energy.

#### 2.2.5. Lipophilic Antioxidants

The lipophilic antioxidants *β*-carotene, lycopene, and chlorophyll a and b were determined using dried and powdered leaf samples (150 mg) vigorously stirred in 10 mL of an acetone-hexane mixture (4 : 6, *v*/*v*) for 1 min and filtered through a Whatman® Grade 4 qualitative filter paper. The absorbance of the filtrate was measured at 453, 505, 645, and 663 nm. The *β*-carotene, lycopene, and chlorophyll a and b contents were calculated using the following equations: *β*-carotene (mg/100 mL) = 0.216 × A663–1.220 × A645 − 0.304 × A505 + 0.452 × A453; lycopene (mg/100 mL) = −0.0458 × A663 + 0.204 × A645 + 0.304 × A505 − 0.0452 × A453; chlorophyll a (mg/100 mL) = 0.99 × A663 − 0.0989 × A645; and chlorophyll b (mg/100 mL) = −0.328 × A663 + 1.77 × A645. The results are expressed as mg/100 g of sample [23].

#### 2.2.6. Fatty Acid Composition

The total lipids extracted from dried and powdered leaf samples of *H. speciosa* were subjected to transesterification with 2.5 mL of methanol, 1.25 mL of sulfuric acid, and 1.25 mL of toluene (2 : 1 : 1 ratio (*v*/*v*)) for at least 12 h in a water bath at 50°C and 160 rpm. After this period, 3 mL of deionized water and 3 mL of diethyl ether were added. The mixture was stirred and left to stand until phase separation. The upper phase of fatty acid methyl esters (FAMEs) was recovered using an anhydrous sodium sulfate microcolumn and transferred to a glass vial using a 0.2 *μ*m Millipore nylon filter before injection into the device [24]. The sample FAMEs were determined by gas chromatography with flame-ionization detection (GC-FID) in a capillary column of the DANI GC 1000 chromatograph (Izasa, Barcelona, Spain), which is equipped with a split/splitless injector, a flame ionization detector, and a Macherey-Nagel column (30 m × 0.32 mm ID × 0.25 *μ*m d_f_). The initial temperature of the column was 50°C for 2 min, followed by a 30°C/min increase until 125°C, a 5°C/min increase until 160°C, a 20°C/min increase to 180°C, and a 3°C/min increase until 200°C. Lastly, the temperature was increased at 20°C/min until the limit of 220°C, where it was held for 15 min. The total analysis time was 35.16 min. The gas (hydrogen) was maintained at a flow rate of 4.0 mL/min (0.61 bar) at 50°C. The injection (1 : 40) was performed at 220°C. For each analysis, 1 *μ*L of sample was injected into the GC. The fatty acids were identified by comparing the retention times of the peaks of standard FAMEs (Supelco 37 Component FAME mix) with those of the sample FAMEs. The results were processed using CSW 1.7 software (DataApex 1.7, Prague, Czech Republic), and the data are expressed as the relative percentage of each fatty acid.

### 2.3. Microbiological Tests

The following parameters were assessed: mesophilic microorganisms, molds, yeasts, total coliforms, *Escherichia coli,* sulphite-reducing *Clostridium* spores, and *Salmonella* spp. All microbiological tests were performed in triplicate.

#### 2.3.1. Sample Preparation

The samples were prepared as previously described by Gomes et al. [25]. For this, 25 g of dried and powdered leaves of *H. speciosa* were aseptically weighed and homogenized using Stomacher Lab-Blender (Seward type 400, London, UK) for 3 minutes with 225 mL of sterile peptone physiological saline solution (0.1% peptone + 0.85% NaCl in sterile deionized water, pH = 7.0 ± 0.05). After 24 hours, serial dilutions were prepared from this homogenate (initial dilution) with the same sterile diluents that were used for quantification of the different microorganisms.

#### 2.3.2. Total Mesophilic Count

The mesophilic aerobic microorganisms were counted by adding 1 mL of each dilution of the leaves of *H. speciosa* to plate count agar (PCA) media (Himedia, Mumbai, India), in accordance with Portuguese law 3788 [26]. Microbial counts are expressed as colony-forming units per gram (cfu/g) of sample.

#### 2.3.3. Mold and Yeast Counts

Molds and yeasts were counted by adding 1 mL of the dilution of the leaves of *H. speciosa* in yeast peptone dextrose agar (PDA) media (Himedia, Mumbai, India) incubated at 25°C for 5 days [27]. Microbial counts are expressed as colony-forming units per gram (cfu/g) of sample.

#### 2.3.4. *Staphylococcus aureus* Count

*Staphylococcus aureus* (*S. aureus*) was quantified according to protocol NP 4400-1 [28]. Serial dilutions of the leaves of *H. speciosa* were inoculated in Baird-Parker broth with egg yolk tellurite emulsion (Himedia) for 24 h (37°C). Subsequently, three to five typical colonies were selected to control for the presence of coagulase and catalase. Microbial counts are expressed as cfu/g of sample.

#### 2.3.5. Total Coliform and *Escherichia coli* Counts

The total coliform and *Escherichia coli* (*E. coli*) were counted using the SimPlate method [29], according to the manufacturer's instructions and procedures.

For this, 1 mL aliquots of the serial dilutions of the leaves of *H. speciosa* were mixed with 9 mL of a blue solution supplied by the manufacturer and dispensed into the center of the SimPlate device. The SimPlate has undergone slight rotations to disperse the sample and remove the air bubbles. Samples were incubated at 37 ± 1°C for 24 to 48 h. The positive count of total coliforms was based on the change in color from blue to pink, and the positive count of *E. coli* was based on the change in color and UV fluorescence. Coliforms and *E. coli* were quantified by counting the number of positive wells, followed by the correlation with the conversion table, and the results are expressed as cfu/g of sample.

#### 2.3.6. Enumeration of Sulphite-Reducing *Clostridium* Spores

To the enumeration of *sulphite-*reducing *Clostridium* spores, 0.1, 1, 5, and 10 mL aliquots of the initial dilution were added to empty tubes, which were heated to 80°C for 5 min and incubated with iron sulphite agar (ISA) media [30] in anaerobic conditions at 37°C for 5 days. The results are determined based on the enumeration of sulphite-reducing *Clostridium* spores in 0.01 g of sample and expressed as cfu/g of sample.

#### 2.3.7. *Salmonella* spp. Detection

*Salmonella* spp. were detected using the immunodiffusion 1-2 test [29]. The test was performed by adding 100 *μ*L of sample (initial dilution) preenriched in buffered peptone water and 50 *μ*L of specific antibody included in the test kit. To confirm immunoband formation, the test samples were read after 16 to 20 h of incubation at 37°C.

### 2.4. Antioxidant Activity Assessment

#### 2.4.1. Preparation of Human Erythrocyte Suspensions

The protocol of human peripheral blood collection was approved by the Research Ethics Committee (Comitê de Ética em Pesquisa (CEP)) of the University Centre of the Grande Dourados (Centro Universitário da Grande Dourados), Brazil (process CEP number 123/12). Peripheral blood was collected from healthy human donors after they signed the informed consent form. The samples were transferred into tubes with sodium citrate as an anticoagulant and centrifuged at 1500 rpm for 10 min at 4°C to separate the hemocomponents. Blood plasma and the thin layer of leukocytes were removed. The erythrocytes were washed three times with phosphate-buffered saline (PBS, pH = 7.4) and centrifuged at 1500 rpm for 10 min at 4°C in each washing. Lastly, a 2% hematocrit suspension was prepared in PBS.

#### 2.4.2. Antihemolytic Activity

The assays were conducted with an erythrocyte suspension (2%) preincubated at 37°C for 30 minutes with 20 *μ*g/mL EEHS or with the flavonoid rutin, catechin, or isoquercetin individually, solubilized in dimethyl sulfoxide (DMSO; final concentration up to 0.08%). After this period, 50 mM 2,2′-azobis(2-amidinopropane) dihydrochloride (AAPH) solution was added to the groups subjected to hemolysis induction. This mixture was incubated at 37°C for 4 h with frequent stirring. Erythrocytes incubated with PBS, the extract, and flavonoids only were used as negative controls. After every 60 min of incubation, the samples were centrifuged at 4000 rpm for 10 min, and an aliquot was collected from the supernatant, followed by dilution in PBS. Then, the samples were read at 545 nm. The percentage of hemolysis was calculated using the formula *A*/*B* × 100, where *A* represents the sample absorbance and *B* represents the total hemolysis (erythrocytes incubated in distilled water). The assays were performed in triplicate. Percentage values were set for the third hour of incubation. The percentage of inhibition of hemolysis as a function of the sample concentration was also determined from the concentration-response curve.

### 2.5. Antimutagenic Activity

The antimutagenic activity was assessed using the Ames test, with some modifications [31]. Two strains were used for the assay, *Salmonella typhimurium* TA98 and TA100, genetically modified with a mutation in the histidine operator gene, which causes the strains to grow in a histidine-dependent manner. The activities of the direct mutagens sodium azide (SAZ) and 4-nitroquinoline 1-oxide (4NQO) and the indirect mutagen aflatoxin (AFB1) were assessed. The rates of inhibition of the mutagenic activity induced by the EEHS (5–15 *μ*g/mL) and flavonoids (0.1–0.5 *μ*g/mL) were calculated using the following equation: rate of inhibition (%) = (*A* − *B*)/*A* × 100, where *A* is the number of revertants in the presence of mutagens and *B* is the number of revertants in the presence of mutagens and samples after subtracting the number of spontaneous revertants. Values > 80% viable cells were considered nontoxic compared with the negative control viability. The antimutagenic effect was considered moderate when the inhibitory effect ranged from 25 to 40% and high when it was greater than 40%. Effects lower than 25% were considered irrelevant and were disregarded.

### 2.6. Enzyme-Inhibiting Activity Assessment

#### 2.6.1. Cholinesterase-Inhibiting Activity

The acetylthiocholinesterase- (AChE-) and butyrylthiocholinesterase- (BChE-) inhibiting activities were assessed based on the spectrophotometric method described by Senol et al. [32] using acetylthiocholine and butyrylthiocholine as substrates. Aliquots of 50 *μ*L of the EEHS or flavonoids (200–700 *μ*g/mL) diluted in EtOH/buffered phosphate (0.1 mM, pH = 8) were incubated with 25 *μ*L acetylcholinesterase (AChE) from *Electrophorus electricus* (Type-VI, EC 3.1.1.7, Sigma-Aldrich, USA) and 125 *μ*L of 5,5′-dithiobis (2-nitrobenzoic acid) (0.01 M) (DTNB, Sigma-Aldrich, USA) for 15 minutes at 25°C. The same procedure was performed to evaluate the activity of butyrylcholinesterase (BChE) from horse serum (EC 3.1.1.8, Sigma-Aldrich, USA). Subsequently, the reactions were initiated with the addition of 25 *μ*L of the substrates acetylthiocholine iodide and butyrylthiocholine chloride (0.075 M, Sigma-Aldrich, USA), respectively, diluted in EtOH/phosphate buffer (0.1 mM, pH = 8). The hydrolysis of substrates was monitored by the formation of the yellow 5-thio-2-nitrobenzoate anion as a result of the reaction with the 5,5′-dithiobis(2-nitrobenzoic acid) (0.01 M) (DTNB, Sigma-Aldrich, EUA) at 412 nm. All tests were performed in triplicate. Eserine (0.001–0.01 *μ*g/mL) (Sigma-Aldrich, USA) was used as a reference compound. The percentages of AChE/BChE enzyme inhibition were determined comparing the reaction rates of the samples compared with the blank (ethanol in phosphate buffer (0.1 mM, pH = 8)) using the following formula: (*E* − *S*)/*E* × 100, where *E* is the enzymatic activity without test samples and *S* is the enzymatic activity with test samples. The IC_50_ values were calculated.

#### 2.6.2. Tyrosinase-Inhibiting Activity

The tyrosinase-inhibiting activity was assessed using the method described by Orhan and Khan [33], with some modifications. Aliquots of 25 *μ*L of the EEHS or flavonoids (50–1000 *μ*g/mL) were mixed with 40 *μ*L of the tyrosinase solution (EC 1.14.1.8.1, 30 U mushroom tyrosinase, Sigma-Aldrich, 200 U/mL) and 100 *μ*L of buffer phosphate solution (pH 6.8) in a 96-well microplate, incubated for 15 min at 37°C. Subsequently, 40 *μ*L of L-DOPA (3,4-dihydroxy-L-phenylalanine) was added to the mixture which was incubated for 10 min at 37°C. The reading of the absorbance was performed at 492 nm. Kojic acid (2.5–20 *μ*g/mL) was used as the reference compound.

All tests were performed in triplicate. The percentage of tyrosinase inhibition was calculated using the following formula: % inhibition = [(blank absorbance − sample absorbance)/blank absorbance] × 100. The IC_50_ values were calculated.

#### 2.6.3. Hyaluronidase-Inhibiting Activity

The assay was performed as described by Ling et al. [34], with some modifications. For this, a mixture of 80 U of the enzyme hyaluronidase in 100 *μ*L of 20 mM sodium phosphate buffer and 25 *μ*L of EEHS or flavonoids (35 *μ*g/mL) dissolved in 25% (*v*/*v*) DMSO was incubated for 10 min at 37°C. Afterwards, 100 *μ*L of hyaluronic acid (0.03% in 300 mM sodium phosphate buffer, pH 5.35) was added and incubated for 45 min at 37°C. The undigested hyaluronic acid was precipitated with 1 mL of acidic albumin solution (0.1% bovine albumin in 24 mM sodium acetate and 79 mM acetic acid at pH 3.75). Posteriorly, the samples were incubated at room temperature for 10 min. The reading of the absorbance was performed at 600 nm. Absorbance in the absence of enzyme was used as a reference for maximal inhibition. Epigallocatechin (35 *μ*g/mL) was used as the reference compound. The tests were performed in triplicate. The inhibitory activity of the test sample was calculated using the following formula: inhibition (%) = *A*_t_ (test sample)/*A*_ref_ (reference sample) × 100.

#### 2.6.4. *α*-Amylase-Inhibiting Activity

The *α*-amylase-inhibiting activity was assessed as described by Gao et al. [35]. Blue starch (Sigma-Aldrich; 2.0 mg) was used as the substrate in 50 mM Tri-HCl buffer at a pH of 6.9 containing 10 mM CaCl_2_ and boiled for 5 min at 100°C. Then, the starch azure solution was preincubated for 10 min at 37 C. The samples of the EEHS or flavonoids (20–100 *μ*g/mL) were dissolved in 25% DMSO, and 200 *μ*L of porcine pancreatic a-amylase solution (Sigma-Aldrich, A-6255; 2.0 U/mL; 50 mM Tri-HCl buffer containing 10 mM CaCl_2_, pH 6.9) was added into each assay. The reaction was carried out at 37°C for 10 min and stopped by adding 0.5 mL of 50% acetic acid. The reaction mixture was then centrifuged at 2000 rpm for 5 min at 4°C. The absorbance of the supernatant at 595 nm was measured. Acarbose (20–100 *μ*g/mL) was used as the reference compound. The tests were performed in triplicate. The *α*-amylase-inhibiting activity was calculated using the following formula: %inhibition = (*A*_c+_) − (*A*_c−_) − (*A*_s_ − *A*_b_)/(*A*_c+_) − (*A*_c−_) × 100, where *A*_c+_ is the activity in the presence of 100% enzyme (solvent with enzyme), *A*_c−_ is the activity in the presence of 0% enzyme (solvent without enzyme), *A*_s_ is the enzyme-inhibiting activity of the test sample (sample with enzyme), and *A*_b_ is the blank (test sample without enzyme). The IC_50_ values were calculated.

#### 2.6.5. *α*-Glucosidase-Inhibiting Activity

The *α*-glucosidase-inhibiting activity was assessed according to Mayur et al. [36], with minor modifications. The reaction mixture contained 50 *μ*L of 0.1 M phosphate buffer (pH 7.0), 25 *μ*L of 0.5 mM 4-nitrophenyl *α*-D-glucopyranoside, 10 *μ*L of the EEHS or flavonoids (25–85 *μ*g/mL), and 25 *μ*L of *α*-glucosidase solution (0.2 unit/mL). The mixture was then incubated at 37°C for 30 min, and the reaction was terminated by adding 100 *μ*L of 0.2 M sodium carbonate solution. The enzymatic hydrolysis of substrate was monitored through the amount of p-nitrophenol released in the reaction mixture at 410 nm. Individual blanks were prepared for correcting background absorbance, being the enzymes replaced by 0.1 M phosphate buffer (pH 7.0). In controls, the plant extract or flavonoids were replaced by methanol. Acarbose (25–85 *μ*g/mL) was used as a positive control. The inhibition percentage of *α*-glucosidase was calculated through the formula: % inhibition = [1 − (sample absorbance/control absorbance)] × 100. The IC50 values were compared; analyses were carried out in triplicate.

#### 2.6.6. Lipase-Inhibiting Activity

The porcine pancreatic lipase (PPL) (type II-inhibiting, Sigma-Aldrich) activity was assessed using the spectrophotometric method described by Roh and Jung [37]. EEHS or flavonoids (final concentrations of 2.5–35 *μ*g/mL) were preincubated with PPL (1 mg/mL) for 1 h in a potassium phosphate buffer (0.1 mM, pH 7.2, 0.1% Tween 80) at 30°C before assaying the PPL activity. 0.1 *μ*L p-nitrofenil Butirato (Sigma-Aldrich) was then added as a substrate. After incubation at 30°C for 5 min, the amount of p-nitrophenol released in the reaction was measured at 405 nm using a UV-visible spectrophotometer. The activity of the negative control (DMSO) was also evaluated with and without an inhibitor. All tests were performed in triplicate. Orlistat (0.003–0.01 *μ*g/mL) was used as the reference compound. The PPL-inhibiting activity was calculated using the following formula: inhibitory activity (%) = 100 − ((*B* − *b*)/(*A* − *a*) × 100), where *A* is the activity without an inhibitor, *a* is the negative control without an inhibitor, *B* is the activity with an inhibitor, and *b* is the negative control with an inhibitor. The IC_50_ values were calculated.

### 2.7. In Vivo Studies

#### 2.7.1. Animals

All experimental procedures with animals were submitted to and approved by the Ethics Committee on Animal Use of the University of Brasília (permission-UnBDOC number 47926/2010) and conducted in accordance with the norms of the National Council for the Control of Animal Experimentation (Conselho Nacional de Experimentação Animal (CONCEA)). 120-day-old adult male Wistar rats were obtained from the animal house of the School of Biological and Environmental Sciences, Federal University of Grande Dourados, Mato Grosso do Sul State, Brazil. The animals were kept in polyethylene boxes at 22 ± 2°C and in a controlled light-dark cycle (12/12 h). All animals were fed ad libitum.

#### 2.7.2. Oral Glucose Tolerance Test in Normoglycemic Wistar Rats

The oral glucose tolerance test (OGTT) was performed after 12 hours of fasting in adult 120-day-old male normoglycemic Wistar rats. The animals were challenged with glucose overload by oral gavage (2 g/kg body mass). After 30 minutes, the vehicle control dimethyl sulfoxide (6% DMSO) and EEHS (200 and 400 mg/kg body mass) treatments were performed. Rat glycemia was measured at 0, 30, 60, 120, and 180 min after delivering the glucose overload according to Aragão et al. [38] using the Accu-Chek Active (Roche) blood glucose meter and disposable strips.

#### 2.7.3. Diabetes Induction

Diabetes was induced in adult 120-day-old male Wistar rats with a single intraperitoneal injection of alloxan monohydrate (120 mg/kg body mass) dissolved in 0.9% sterile saline, as described by Aragão et al. [38], with some modifications. Blood glucose levels were determined 96 h after the induction of diabetes using a portable Accu-Chek (Abbott) blood glucose meter and disposable strips. The animals with hyperglycemia (blood glucose levels higher than 200 mg/dL) were separated for further study.

#### 2.7.4. Glycemic Assessment in Diabetic Wistar Rats

The treatment and glycemic assessment of diabetic male Wistar rats were performed according to Aragão et al. [38], with some modifications. The blood glucose levels of diabetic rats submitted to acute and chronic treatment (28 days) were measured during fasting at time zero, and they were subsequently administered with the doses of vehicle control 6% DMSO, EEHS (200 mg/kg body mass), and metformin (120 mg/kg body mass). The blood glucose levels of the rats were measured at times of 0, 30, 60, 120, and 180 min using the Accu-Chek Active (Roche) blood glucose meter and disposable strips.

### 2.8. Statistical Analysis

The data are expressed as the mean ± standard error of the mean (SEM) and were analyzed to assess significant differences between groups. One-way analysis of variance was used for the total composition of FAMEs as well as antihemolytic, enzyme-inhibiting, and antimutagenic activities. Differences were determined using the multiple comparison Tukey test for the results of the total composition of FAMEs and antihemolytic activity. Dunnett's multiple comparison test was used to assess differences in enzyme inhibition and antimutagenicity assays. The *t*-test was used in the *in vivo* experiments to assess differences between groups. All data were obtained using the Software GraphPad Prism 5. Results were considered significant when ^∗^*P* < 0.05 or ^†^*P* < 0.05, ^∗∗^*P* < 0.01, and ^∗∗∗^*P* < 0.001 or ^###^*P* < 0.001.

## 3. Results

### 3.1. Physicochemical Parameters

Physicochemical parameters, including the mineral (ash), protein, total lipid, and carbohydrate contents as well as the energy value of *H. speciosa* leaves, are outlined in Table 1.

### 3.2. Lipophilic Antioxidants

Chlorophyll a and *β*-carotene were the predominant antioxidants, with 1.33 ± 0.03 and 0.55 ± 0.01 mg/100 g of sample, respectively. Conversely, the concentration of chlorophyll b was 0.42 ± 0.02 mg/100 g, and the concentration of lycopene was 0.070 ± 0.01 mg/100 g.

### 3.3. Profile of Fatty Acid Methyl Esters

**Figure 1:**
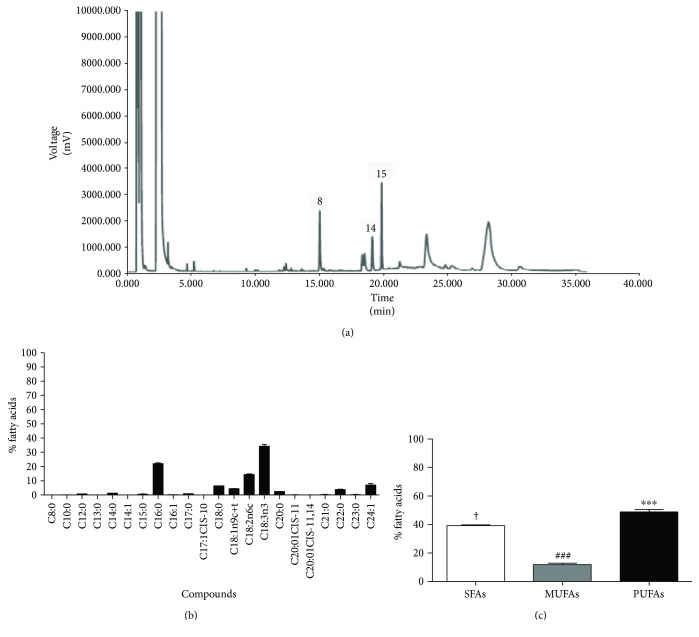
(a) Chromatographic fatty acid profile of *Hancornia speciosa* leaf samples. (b) Chemical composition of fatty acid methyl esters (FAMEs): caprylic acid (C8:0), capric acid (C10:0), lauric acid (C12:0), tridecanooic acid (C13:0), myristic acid (C14:0), myristoleic acid (C14:1), pentadecanoic (C15:0), palmitic acid (C16:0), palmitoleic acid (C16:1), heptadecanoic acid (C17:0), cis-10-heptadecanoic acid (C17:1), stearic acid (C18:0), oleic acid (C18:1n9c+t), linoleic acid (C18:2n6c), *α*-linolenic acid (C18:3n3), arachidic acid (C20:0), cis-11-eicosenoic acid (C20:1), cis-11,14-eicosadienoic acid (C20:2), heneicosanoic acid (C21:0), behenic acid (C22:0), tricosanoic acid (C23:0), and nervonic acid (C24:1). (c) Total composition of saturated fatty acids (SFAs), monounsaturated fatty acids (MUFAs), and polyunsaturated fatty acids (PUFAs) of *Hancornia speciosa* leaf samples. Values are expressed as the mean ± SEM of three independent experiments performed in duplicate. ^†^*P* < 0.05 compared with the SFA group versus the PUFA group; ^###^*P* < 0.001 compared with the MUFA group versus the PUFA and SFA groups; and ^∗∗∗^*P* < 0.001 compared with the PUFA group versus the MUFA groups.

After total lipid extraction from *H. speciosa* leaves, the profiles of FAMEs were analyzed by gas chromatography (Figure 1(a)). The *α*-linolenic acid (C18:3*n*3) was the predominant fatty acid identified in the sample, followed by palmitic (C16:0) and linoleic (C18:2*n*6) acids. In addition to these three fatty acids, 19 other compounds were identified and quantified (Figure 1(b)). The sample had a higher percentage of polyunsaturated fatty acids (PUFAs) (48.82%), followed by saturated fatty acids (SFAs) (39.19%) and monounsaturated fatty acids (MUFAs) (11.86%) (Figure 1(c)). The PUFAs/SFAs and *n*-6/*n*-3 ratios assessed were 1.25 and 3.37, respectively.

### 3.4. Microbiological Quality and Safety

The results from the microbiological quality and safety analysis of *H. speciosa* leaves are outlined in Table 2.

### 3.5. Antihemolytic Activity

**Figure 2:**
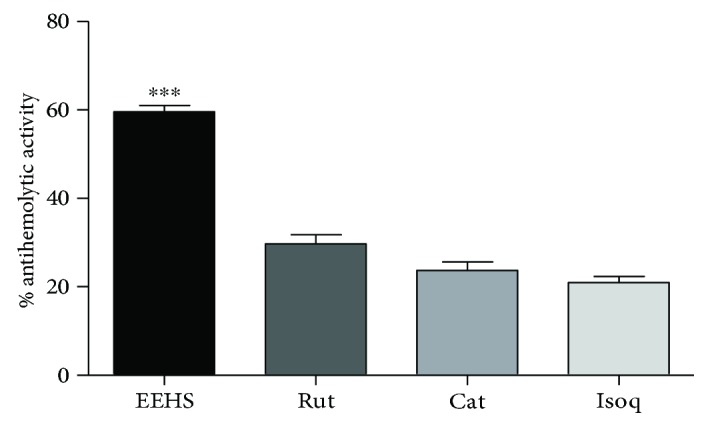
Antihemolytic activities of the ethanolic extract from *Hancornia speciosa* leaves (EEHS) (20 *μ*g/mL) and the flavonoids rutin (Rut), catechin (Cat), and isoquercetin (Isoq) (20 *μ*g/mL) against 50 mM AAPH-induced hemolysis. Values are expressed as the mean ± SEM of experiments performed in triplicate. ^∗∗∗^*P* < 0.001 when the EEHS group is compared with the rutin, catechin, and isoquercetin groups.

Figure 2 shows the results of the protective activities of the EEHS (59.59 ± 1.42%), rutin (29.67 ± 2.11%), catechin (23.67 ± 1.97%), and isoquercetin (20.97 ± 1.38%) against AAPH-induced hemolysis.

### 3.6. Antimutagenic Activity

The results from the study of the antimutagenic potentials of the EEHS, rutin, catechin, and isoquercetin are outlined in Table 3. In all of the EEHS concentrations tested (5–15 *μ*g/mL), a high antimutagenic activity was observed in the *Salmonella typhimurium* TA98 and TA100 strains exposed to different mutagens with (S9+) and without (S9−) metabolic activation. Among the flavonoids, rutin (0.1–0.25 *μ*g/mL) showed moderate antimutagenic activity in the *Salmonella typhimurium* TA98 strain without metabolic activation.

### 3.7. Enzyme-Inhibiting Activity

#### 3.7.1. Cholinesterase-Inhibiting Activity

Table 4 shows that the EEHS had higher AChE-inhibiting activity than isoquercetin (2.0 times), rutin (1.7 times), and catechin (1.5 times) and lower activity than eserine (reference AChE inhibitor). The EEHS showed higher BChE-inhibiting activity than isoquercetin (1.8 times), catechin (1.6 times), and rutin (1.4 times) and lower activity than eserine (reference BChE inhibitor) (Table 4).

#### 3.7.2. Tyrosinase-Inhibiting Activity

The EEHS showed 4.3-, 3.8-, and 3.7-fold higher tyrosinase-inhibiting activity than catechin, isoquercetin, and rutin, respectively (Table 4). The tyrosinase-inhibiting activities of all compounds were lower than that of the reference inhibitor kojic acid (Table 4).

#### 3.7.3. Hyaluronidase-Inhibiting Activity

The EEHS showed 1.8-fold higher hyaluronidase-inhibiting activity than rutin and catechin and 2.6-fold higher activity than isoquercetin (Table 4). However, the hyaluronidase-inhibiting activities of the EEHS and flavonoids were lower than that of the reference compound, epigallocatechin (EGC) (Table 4).

#### 3.7.4. Pancreatic Lipase-Inhibiting Activity

Table 4 shows that the EEHS had higher pancreatic lipase-inhibiting activity than isoquercetin (8.1 times), catechin (7.4 times), and rutin (5.2 times) and lower activity than that of orlistat (reference inhibitor of pancreatic lipase).

#### 3.7.5. *α*-Amylase- and *α*-Glucosidase-Inhibiting Activities

Table 4 shows that the EEHS had higher *α*-amylase-inhibiting activity than rutin (2.2 times), isoquercetin (2.0 times), catechin (1.4 times) and the reference inhibitor acarbose (1.9 times). The assessment of the *α*-amylase-inhibiting activity of flavonoids in comparison with the reference compound acarbose showed that catechin had a higher activity than acarbose (Table 4). The EEHS showed higher *α*-glucosidase-inhibiting activity than rutin (2.4 times), catechin (2.2 times), isoquercetin (1.7 times), and acarbose (1.6 times) (Table 4).

### 3.8. *In Vivo* Studies

#### 3.8.1. Oral Glucose Tolerance Test in Normoglycemic Wistar Rats

**Figure 3:**
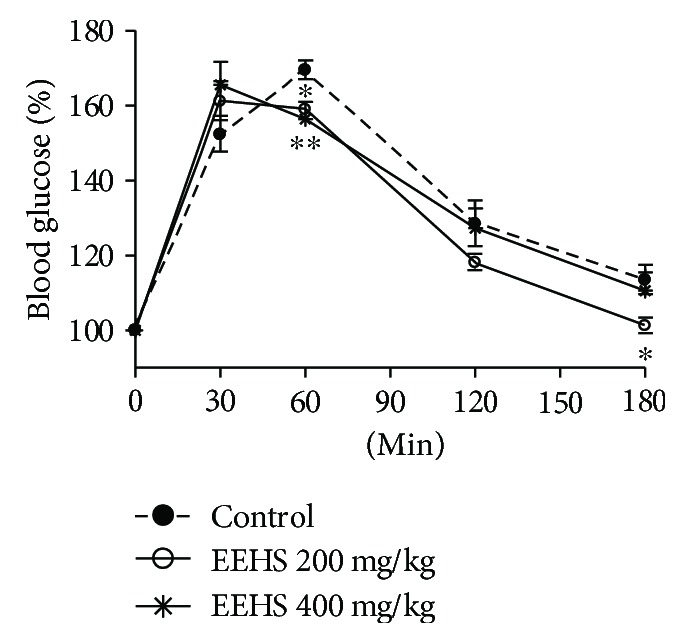
Oral glucose tolerance test in normoglycemic Wistar rats subjected to treatment with the control and ethanolic extract of *Hancornia speciosa* leaves (EEHS) at 200 and 400 mg/kg. Times assessed: 0, 30, 60, 120, and 180 min. Values are expressed as the mean ± SEM, *n* = 5. ^∗∗^*P* < 0.01 and ^∗^*P* < 0.05 when comparing treatment groups with the control group.

Figure 3 shows that the normoglycemic rats challenged with glucose overload show significantly decreased blood glucose levels when treated with the EEHS (200 mg/kg).

#### 3.8.2. Glycemic Assessment in Diabetic Wistar Rats

**Figure 4:**
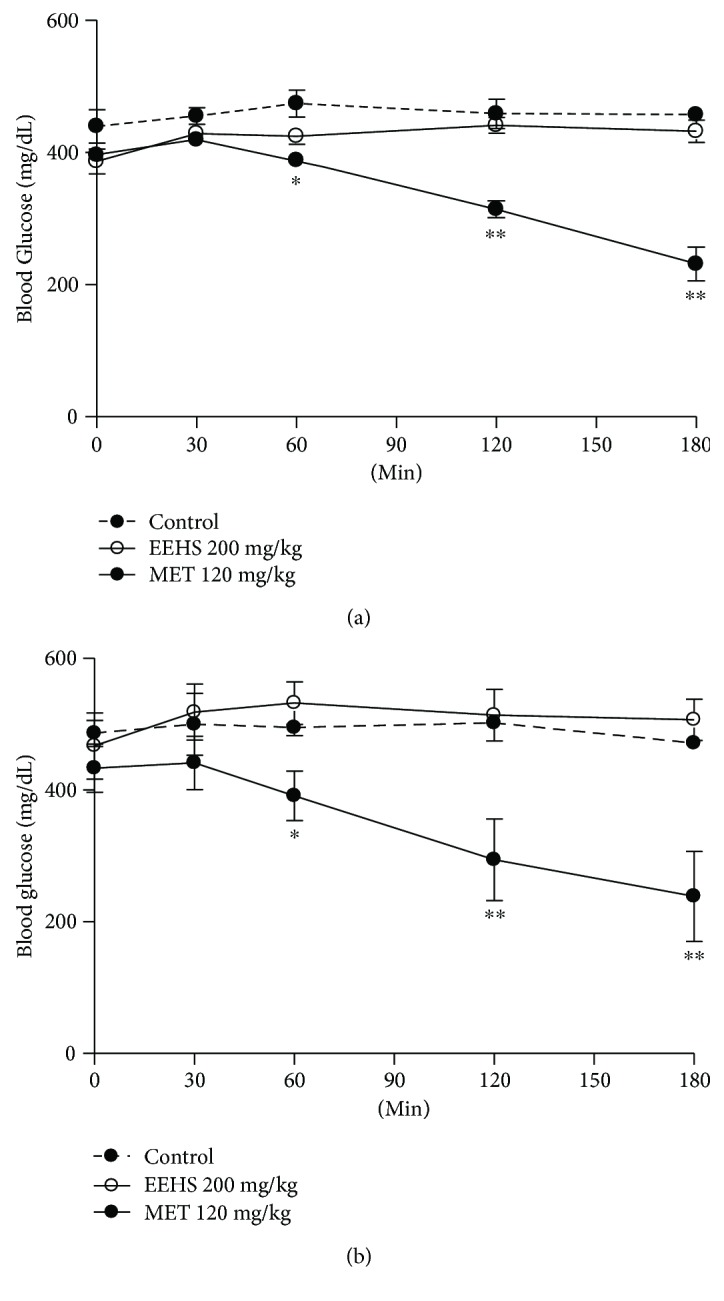
Glycemic curve without glucose overload in diabetic Wistar rats subjected to acute (a) and chronic (b) treatments with the control, EEHS (200 mg/kg) and metformin (MET, 120 mg/kg). Times tested: 0, 30, 60, 120, and 180 min. Values are expressed as the mean ± SEM, *n* = 5. ^∗∗^*P* < 0.01 and ^∗^*P* < 0.05 when comparing treatment groups with the control group.

In the assay with diabetic rats with glycemia higher than 400 mg/dL and without glucose overload, no decreases in the blood glucose levels were observed after acute (Figure 4(a)) and chronic (Figure 4(b)) treatments with the EEHS (200 mg/kg). Only the experimental group metformin (MET, 120 mg/kg) showed decreased blood glucose levels (Figures 4(a) and 4(b)).

## 4. Discussion

Brazil has different biomes with high biological diversity and native species, which are often used as medicinal plants. Studying the pharmacological properties of these plants used in traditional medicine ensures its scientific record and strengthens the traditional knowledge accumulated over centuries. Among these plants, *H. speciosa* has been described as a highly versatile species. This plant is traditionally used to treat various diseases and has a high economic and biotechnological potential for drug development because its leaves and other parts are sold as tea [4, 39].

Despite the proven therapeutic effect, the increase in the consumption of natural products has become a public health problem because low-quality products without validation of raw material safety and efficiency may be purchased [3]. Natural products may contain a large number of fungi and bacteria; these microorganisms are usually from the soil, which is the natural microbiota or even introduced during inappropriate handling of harvest, drying, and storage of these products (WHO, 2007). In this sense, several studies have been carried out with the purpose of assuring the quality and microbiological safety of medicinal plants [40–42]. Although the European Community legislation does not have defined microbiological standards for aromatic dry plants, the World Health Organization (WHO), the European Spice Association (ESA), and the Codex Code of Hygienic Practice specify that aromatic dry plants need to have acceptable microbial contamination levels and absence of pathogenic microorganisms, such as *Salmonella* sp. and *Clostridium* sp. [2, 43, 44]. Thus, this study aimed to ensure that the raw material used is of high quality and safe for consumption by testing the microbiological quality and safety of *H. speciosa* leaves, which are in accordance with current standards established by the World Health Organization for products intended for human consumption [2].

After the initial quality and safety assessment as well as physicochemical analysis, including the quantification of the total lipids extracted from *H. speciosa* leaves, different fatty acids were identified. Among the fatty acids, *α*-linolenic acid (polyunsaturated omega-3), considered an essential fatty acid only obtained from the diet [45], was the predominant fatty acid found in *H. speciosa* leaves, followed by palmitic (saturated) and linoleic (polyunsaturated omega-6) acids. Moreover, the PUFAs/SFAs and *n*-6/*n*-3 fatty acid ratios calculated indicate good nutritional quality, including health benefits [46]. *α*-Linolenic acid cannot be synthesized by humans; however, dietary *α*-linolenic acid is the precursor of eicosapentaenoic and docosahexaenoic acids, which are anti-inflammatory eicosanoids [47, 48].

Furthermore, the quantification of natural antioxidants, including lipophilic antioxidants as well as the total phenols and flavonoids in natural products, provides key parameters to assess the quality and biological potential of herbal products [49]. In this study, the lipophilic antioxidants chlorophyll a and b, *β*-carotene, and lycopene were identified and quantified in *H. speciosa* leaves. The main carotenoids include the hydrocarbons *β*-carotene and lycopene [50]. The great interest in these nutrients results from their biological and physiological functions [51]. Some carotenoids, in addition to vitamin A (retinol) precursors, have antioxidant properties and improve the immune response [52]. Moreover, a previous study by Santos et al. [4] with the EEHS reported higher concentrations of total phenols than those described for *H. speciosa* fruit extracts [53].

Phenolic compounds are hydrogen donors capable of directly removing free radicals and reducing oxidative damage [4, 54, 55]. Extracts from medicinal plants with phenols and flavonoids, including *H. speciosa*, are described for their ability to activate endogenous antioxidant systems and to inhibit lipid peroxidation in human erythrocytes [4, 56, 57]. Accordingly, the flavonoids rutin, catechin, and isoquercetin, previously identified by Santos et al. [4] in the EEHS, showed antioxidant activity in this study, which was confirmed by the protection against human erythrocyte hemolysis resulting from lipid peroxidation. However, the results showed a possible synergistic effect between components of the extract because the EEHS showed a higher antihemolytic activity than the flavonoids alone. Peroxyl radicals promote hemolysis by oxidizing lipids and proteins of the cell membrane [58]. *In vitro* and *in vivo* studies show that natural extracts and flavonoids have antioxidant activity by inhibiting lipid peroxidation in the erythrocytes and cardiomyocytes of rats subjected to oxidative stress [4, 59, 60]. The protection against lipid peroxidation promoted by the test samples may be associated with the direct peroxyl radical scavenging activity or with the activation of enzymatic antioxidant mechanisms, such as glutathione, catalase, and superoxide dismutase [56, 58].

Accordingly, endogenous intracellular antioxidant pathways naturally protect the human body from the frequent contact with mutagenic agents. These structures stabilize highly reactive species that damage deoxyribonucleic acid (DNA) [61]. However, natural defense mechanisms may not suffice, thus highlighting the importance of investigating exogenous antioxidant compounds with antimutagenic activity [62].

The Ames test has been recommended to assess the antimutagenic effects of several natural molecules, including plant compounds. This assay can detect frameshift or missense mutations [63]. The EEHS showed antimutagenic activity when *Salmonella typhimurium* TA98 and TA100 strains were exposed to the direct mutagens 4-NO, a substitution agent that primarily acts on guanine (G) residues, inducing transitions from guanine-cytosine (GC) to adenine-thymine (AT) [64], and SAZ, which induces mutagenesis through the production of the DNA-interacting metabolite L-azidoalanine, inducing transitions from GC to AT [65]. Moreover, the EEHS showed antimutagenic activity when *S. typhimurium* TA98 and TA100 strains were exposed to the indirect mutagen AFB1, a toxin that stimulates the release of free radicals, thereby triggering chromosomal aberrations [66]. Among the flavonoids, rutin showed moderate antimutagenic activity when *S. typhimurium* TA98 strains were exposed to 4-NO. A study using the Ames test suggests that flavonoids may show antimutagenic effects through different mechanisms, including scavenging bacterial mutagens, interacting with mutagenic reactive intermediates and affecting microsomal enzymes [67]. However, flavonoids may also show dual concentration-dependent effects, either mutagenic or antimutagenic [68]. Thus, the antimutagenic activity of the extract and rutin could be related to the antioxidant activity because other studies have reported that antioxidant compounds are related to the antimutagenic activity [69, 70].

The ability of flavonoids to reduce oxidative stress is also related to the decreased risk associated with neurodegenerative diseases [71]. In this context, medicinal plants and phenolic derivatives have been recognized for their inhibitory activities against enzymes involved in neurodegenerative diseases [70–72]. Alzheimer's disease is a degenerative and progressive disease and is the form of dementia most commonly found among elderly people [73]. This disease is characterized by the presence of *β*-amyloid plaques with neurofibrillary complications and the degeneration or atrophy of cholinergic neurons [74, 75]. The loss of cholinergic neurons results in the reduced availability of the cholinergic neurotransmitter acetylcholine, leading to impaired cognition in Alzheimer's disease [76]. However, the level of this neurotransmitter can be increased in the brain by inhibiting the activity of cholinesterases, enzymes that break down acetylcholine [77]. In this context, AChE and BChE inhibitors are used to increase the synaptic levels of acetylcholine, which is considered the main disease treatment approach [78]. The increase in acetylcholine levels improves the communication between nerve cells that use it as the only neurotransmitter [79]. Other *in vitro* studies using the flavonoids rutin and quercetin reported findings on the AChE- and BChE-inhibiting activities [72, 80]. *In vivo* phenolic and flavonoid derivatives, such as rutin and quercetin, also show AChE- and BChE-inhibiting activities and improve the cognitive abilities of animals, including those subjected to neurotoxicity [81, 82]. Furthermore, a molecular coupling study has shown the hydrophobic interactions and strong hydrogen bonds between quercetin and the enzymes AChE and BChE, thus proposing this mechanism as the basis for the enzyme-inhibiting activity [83].

The activity of tyrosinase, another enzyme related to neurodegenerative diseases, was inhibited by the *H. speciosa* extract and flavonoids. Tyrosinase is a polyphenol oxidase enzyme involved in the synthesis of melanin (skin and hair) and neuromelanin [84], and it is considered a key target in the search for new drugs against Parkinson's disease [72]. This neurodegenerative disease results from the deficiency of dopaminergic neurons [84]. Studies suggest that tyrosinase plays a role in the formation of reactive species that oxidize dopamine, which triggers the production of more reactive oxygen species leading to neuronal death [85, 86]. Furthermore, the increase in lipid peroxidation in the brains of patients with Parkinson's disease has been reported [87, 88].

In addition to those activities, tyrosinase also plays an enzymatic role in fruit and vegetable browning and skin hyperpigmentation [89]. Accordingly, tyrosinase inhibitors are mainly used as chemical agents to reduce food browning and human skin hyperpigmentation [90, 91]. Although melanogenesis is mainly responsible for skin coloration and the protection of skin against sun-related lesions, abnormal skin hyperpigmentation causes serious esthetic problems [92]. Kojic acid, a chemical product commonly used for skin whitening, has health adverse effects, including cytotoxic and mutagenic effects [93]. Therefore, compounds derived from natural products have become the alternative to those products, including flavonoids, which have stood out as inhibitors of this enzyme. Flavonoids are used to suppress melanogenesis in dermatological diseases associated with skin hyperpigmentation [94]. The assessment of tyrosinase inhibition by the EEHS and flavonoids observed in this study may be related to direct interaction with tyrosinase because flavonoids, including the derivative quercetin, interact with the enzyme forming a flavonoid-copper-enzyme complex, according to Kim et al. [90].

In addition to neurodegenerative diseases and skin hyperpigmentation, inflammatory processes are also involved in the origin of other diseases [75].

In this context, *H. speciosa* has been used in traditional medicine to treat inflammatory diseases [95]. *In vitro* and *in vivo* studies with extracts from the plant and flavonoids isolated from its extracts have shown anti-inflammatory activity through various mechanisms, including the inhibition of the enzymes nitric oxide synthase (iNOS) and cyclooxygenase-2 (COX-2), the proinflammatory transcriptional factor NF-*κ*B, and the inflammatory cytokines interleukin 6 (IL-6) and tumor necrosis factor alpha (TNF-*α*) [5, 6, 96–98].

Many commercially available anti-inflammatory drugs have harmful side effects. Therefore, the search for new therapies is extremely important [98]. Hyaluronidase-inhibiting compounds may be used as anti-inflammatory drugs [99]. The hyaluronidase enzymatic activity not only promotes chemokine and cytokine induction but may also degrade hyaluronic acid [99, 100]. Studies show that the high-molecular-weight hyaluronic acid has an anti-inflammatory activity, whereas its low-molecular-weight degradation products are potent proinflammatory molecules [101]. Hyaluronic acid is the main component of the extracellular matrix and is considered one of the main molecules involved in the tissue regeneration process. The activity of hyaluronic acid and its degradation products have been related to inflammation, cell migration, and angiogenesis through modulation of specific receptors [102]. Some of the consequences of hyaluronic acid degradation resulting from the activity of the enzyme hyaluronidase are bone loss, inflammation, and pain [103].

Another diseased related to inflammation is obesity [104]. Obesity is defined as excess weight [105]. Some of the drug alternatives to treat obesity are related to the inhibition of enzymes involved in fat digestion and absorption [106]. Dietary fat digestion is necessary for its absorption, and such a process occurs through lipid hydrolysis resulting from lipase activity, thereby releasing fatty acids and glycerol [107]. The inhibition of pancreatic lipase, the main enzyme responsible for triglyceride digestion, is a strategy to reduce fat absorption and control weight that may decrease obesity [19]. In this context, many medicinal plants and phenolic derivatives have lipase-inhibiting activity [16–19]. *H. speciosa* is commonly used to treat obesity [108], and in this study, the EEHS showed higher lipase-inhibiting activity in the pancreas than flavonoids and a lower activity to orlistat, a potent commercial inhibitor of this enzyme. These results suggest that the EEHS and the flavonoids tested in this study promote interaction with the lipase enzyme because, as reported by a study, flavonoids, including rutin and quercetin derivatives, interact with lipase forming a flavonoid-enzyme complex, primarily through hydrophobic and hydrophilic bonds, thereby decreasing or inhibiting the activity of the enzyme [109].

Furthermore, obese patients have a high risk of developing diseases, including type 2 diabetes, the most prevalent form of the disease [105, 110]. Type 2 diabetes mellitus is a complex metabolic disease characterized by hyperglycemia resulting from increased insulin resistance [111, 112].

Decreasing postprandial hyperglycemia in patients with type 2 diabetes has been considered the most common therapeutic approach for disease control. Furthermore, this treatment reduces the risk of developing comorbidities and complications, including oxidative stress, obesity, and cardiovascular diseases [113]. This decrease may be achieved by inhibiting the enzymes involved in dietary glucose release, including the enzymes *α*-amylase and *α*-glucosidase [107]. Pancreatic *α*-amylase breaks down starch molecules [114], and intestinal *α*-glucosidase catalyzes the final step of the digestion of starch and disaccharides into glucose for absorption [115]. Therefore, *α*-amylase and *α*-glucosidase inhibitors decrease the postprandial blood glucose levels of diabetic patients [116].

Accordingly, the EEHS, rutin, catechin, and isoquercetin inhibit the enzyme *α*-amylase, and the enzyme-inhibiting activities of the EEHS and catechin are higher than that of the commercial drug acarbose. The EEHS, rutin, catechin, and isoquercetin also showed *α*-glucosidase-inhibiting activity, although only the EEHS-induced *α*-glucosidase-inhibiting activity was higher than that of acarbose. Thus, these results demonstrate the high antidiabetic potential of the extracts and flavonoids tested because they had higher inhibitory activities than the commercial drug sold to treat diabetes. In addition, previous studies with ethanolic leaf extracts from *H. speciosa* and the flavonoid rutin, the main compound present in the EEHS that was tested in this study, have reported *α*-glucosidase-inhibiting activity as well as *in vitro* and *in vivo* control of the blood glucose levels [11]. Thus, the EEHS-induced *α*-amylase and *α*-glucosidase inhibition is a possible mechanism for the reduction of blood glucose levels by the EEHS in animals subjected to glucose overload because diabetic animals showed no improvement in the insulin sensitivity of these animals when compared with treatment with the insulin sensitizer metformin. Therefore, the *in vitro* and *in vivo* findings of this study confirm the role of the EEHS in controlling postprandial hyperglycemia.

Taken together, the results ensure the microbiological quality and safety of *H. speciosa* leaves, and carotenoids and polyunsaturated fatty acids in the leaves were also identified. In addition, the antioxidant, antimutagenic, anti-inflammatory, anti-Alzheimer's disease, anti-Parkinson's disease, antiobesity, and antihyperglycemic activities of the EEHS were identified.

## Figures and Tables

**Table 1:** Physicochemical characterization of *Hancornia speciosa* leaf samples.

Physicochemical characterization	Results/100 g
Ash	2.2 ± 0.0123 g
Protein	14.7 ± 0.1811 g
Lipid	0.14 ± 0.0006 g
Sugars	82.9 ± 0.0174 g
Energy	392.0 ± 0.049 kcal

Values are expressed as the mean ± SEM of experiments performed in triplicate.

**Table 2:** Microbiological analysis of *Hancornia speciosa* leaf samples.

Microbiological analysis	Results
Aerobic mesophiles	6.25 × 10^4^ ± 0.45 cfu/g
Molds and yeast	6.82 × 10^3^ ± 0.18 cfu/g
Total coliforms	12.4 × 10^3^ ± 0.40 cfu/g
*Escherichia coli*	<1 cfu/g
Sulphite-reducing C*lostridia*	Absent
*Salmonella* spp.	Absent
*Staphylococcus aureus*	<10 cfu/g

Values are expressed as the mean ± SEM of experiments performed in triplicate.

**Table 3:** Antimutagenic activities of the ethanolic extract from *Hancornia speciosa* leaves (EEHS), rutin (Rut), catechin (Cat), and isoquercetin (Isoq) in *Salmonella typhimurium* TA98 and TA100 strains.

		S9−		S9+		S9−		S9+	
	*μ*g/mL	TA 98	No revertants (%)	TA 98	No revertants (%)	TA 100	No revertants (%)	TA 100	No revertants (%)
NC		39.0 ± 3.2	—	50.3 ± 3.8	—	63.3 ± 2.6	—	46.7 ± 2.4	—

4-NO	3	198.6 ± 4.6	0	—	—	—	—	—	—

SAZ	3	—	—	—	—	256.7 ± 2.9	0	—	—

AFB1	3	—	—	231.0 ± 6.6	0	—	—	236.3 ± 5.9	0

EEHS	5	35.0 ± 2.1^∗∗∗^	82.3 ± 0.6	18.6 ± 0.9^∗∗∗^	92.3 ± 0.3	95.0 ± 2.9^∗∗∗^	63.0 ± 1.0	75.0 ± 2.9^∗∗∗^	68.0 ± 1.5
	10	11.3 ± 2.3^∗∗∗^	94.0 ± 1.7	8.0 ± 1.1^∗∗∗^	96.7 ± 0.3	70.7 ± 2.3^∗∗∗^	72.3 ± 0.9	57.3 ± 4.3^∗∗∗^	75.7 ± 1.4
	15	7.7 ± 1.20^∗∗∗^	96.0 ± 0.6	5.6 ± 0.3^∗∗∗^	97.6 ± 0.3	44.0 ± 3.8^∗∗∗^	82.7 ± 1.2	36.0 ± 3.6^∗∗∗^	84.7 ± 1.3

Rut	0.1	136.0 ± 5.5^∗∗∗^	31.3 ± 4.1	231.3 ± 4.1	−0.04 ± 0.01	269.7 ± 4.7	−0.05 ± 0.01	254.7 ± 5.2	0.01 ± 0.01
	0.25	148.3 ± 0.9^∗∗∗^	25.3 ± 1.2	248.6 ± 2.0	−0.07 ± 0.01	285.7 ± 2.9	−0.11 ± 0.04	269.3 ± 0.6	−0.05 ± 0.01
	0.5	175.0 ± 2.9	12.3 ± 0.6	>300	—	285.7 ± 0.9	−0.39 ± 0.5	275.3 ± 2.9	0.07 ± 0.01

Cat	0.1	211.0 ± 6.08	−0.06 ± 0.01	240.0 ± 7.1	−0.04 ± 0.01	284.7 ± 1.4	−0.11 ± 0.03	285.0 ± 3.6	−0.11 ± 0.01
	0.25	240.0 ± 7.63	−20.6 ± 2.2	272.3 ± 3.6	−18.0 ± 3.5	289.3 ± 2.2	−0.12 ± 0.02	280.3 ± 4.8	−0.09 ± 0.01
	0.5	>300	—	>300	—	291.0 ± 5.5	−0.06 ± 0.1	284.7 ± 6.8	−0.11 ± 0.02

Isoq	0.1	195.6 ± 5.0	0.01 ± 0.01	201.0 ± 4.3	0.1 ± 0.01	273.3 ± 3.3	−0.06 ± 0.01	281.0 ± 7.4	0.05 ± 0.06
	0.25	>300	—	245.3 ± 6.6	−0.06 ± 0.01	284.3 ± 2.2	−0.31 ± 0.33	279.3 ± 5.8	−0.09 ± 0.02
	0.5	>300	—	>300	—	289.0 ± 2.8	−0.12 ± 0.04	282.3 ± 4.3	−0.37 ± 0.03

*Salmonella typhimurium* TA98 and TA100 strains (CFUs) exposed to the direct mutagens 4-nitroquinoline-1-oxide (4-NO) and sodium azide (SAZ) without metabolic activation (S9−). *Salmonella typhimurium* TA98 and TA100 (CFUs) strains exposed to the indirect mutagen aflatoxin B1 (AFB1) with metabolic activation (S9+, microsomal activation system: microsomal fraction of rat liver homogenate). Values are expressed as the mean ± SEM of the experiment performed in triplicate. ^∗∗∗^*P* < 0.001 when the means of the treated groups are compared with the 4-NO (TA98/without S9), SAZ (TA100/without S9), and AFB1 (TA98 and TA100 with S9) groups. —: unvalued.

**Table 4:** Acetylcholinesterase- (AChE-), butyrylcholinesterase- (BChE-), tyrosinase-, hyaluronidase-, pancreatic lipase-, *α*-amylase-, and *α*-glucosidase-inhibiting activities of the ethanolic extract from *Hancornia speciosa* leaves (EEHS), rutin (Rut), catechin (Cat), and isoquercetin (Isoq).

	AChE	BchE	Tyrosinase	Hyaluronidase	Pancreatic lipase	*α*-Amylase	*α*-Glucosidase
	IC_50_ (*μ*g/mL)	IC_50_ (*μ*g/mL)	IC_50_ (*μ*g/mL)	% (*μ*g/mL)	IC_50_ (*μ*g/mL)	IC_50_ (*μ*g/mL)	IC_50_ (*μ*g/mL)
EEHS	257.2 ± 10.8^∗∗∗^	190.3 ± 9.8^∗∗∗^	159.4 ± 2.4^∗∗∗^	22.5 ± 1.6^∗∗∗^	3.3 ± 0.3^∗∗∗^	35.8 ± 2.9^∗∗∗^	31.6 ± 1.5^∗∗∗^
Rut	437.6 ± 9.9	272.9 ± 8.8	583.2 ± 12.9	12.3 ± 0.9	17.2 ± 0.9	77.9 ± 0.6	74.9 ± 2.3
Cat	379.0 ± 8.4	312.6 ± 6.7	679.2 ± 11.8	12.3 ± 0.8	24.5 ± 0.6	50.3 ± 2.6^∗∗^	68.4 ± 1.6
Isoq	527.8 ± 12.1	342.8 ± 7.6	610.7 ± 7.1	8.8 ± 0.8	26.7 ± 1.6	73.2 ± 1.5	54.9 ± 2.3
Eserine	0.005 ± 0.001	0.037 ± 0.003	—	—	—	—	—
Kojic ac.	—	—	6.5 ± 0.5	—	—	—	—
EGC	—	—	—	97.9 ± 0.8	—	—	—
Orlistat	—	—	—	—	0.016 ± 0.02	—	—
Acarbose	—	—	—	—	—	67.5 ± 1.5	49.7 ± 2.1

Values are expressed as the mean (*μ*g/mL), IC_50_, and percentage ± SEM of experiments performed in triplicate. ^∗∗∗^*P* < 0.001 when comparing the treatment groups with the acarbose control groups, epigallocatechin (EGC). ^∗∗^*P* < 0.01 compared with the catechin group versus the rutin, isoquercetin, and control groups (acarbose). ^∗∗∗^*P* < 0.001 compared with the EEHS group versus the rutin, catechin, isoquercetin, and control groups (eserine, kojic acid, ECG: epigallocatechin, orlistat, or acarbose).

## Abbreviations

EEHS: Ethanolic extract of *H. speciosa* leaves
GC-FID: Gas-liquid chromatography with flame ionization detection
AAPH: 2,2′-Azobis(2-amidinopropane) dihydrochloride
HPLC-DAD-MS/MS: High-performance liquid chromatography with diode-array detection-tandem mass spectrometry
FAMEs: Fatty acid methyl esters
PDA: Yeast peptone dextrose agar
PCA: Plate count agar
ISA: Iron sulphite agar
SAZ: Sodium azide
4NQO: 4-Nitroquinoline 1-oxide
AFB1: Aflatoxin
AChE: Acetylthiocholinesterase
BChE: Butyrylthiocholinesterase
PPL: Porcine pancreatic lipase
OGTT: Oral glucose tolerance test
DMSO: Dimethyl sulfoxide
PUFAs: Polyunsaturated fatty acids
SFAs: Saturated fatty acids
MUFAs: Monounsaturated fatty acids
Rut: Rutin
Cat: Catechin
Isoq: Isoquercetin
DNA: Deoxyribonucleic acid
GC: Guanine-cytosine
AT: Adenine-thymine
iNOS: Nitric oxide synthase
COX-2: Cyclooxygenase-2
IL-6: Interleukin 6
TNF-*α*: Tumor necrosis factor alpha.
